# Biochemical diagnosis of Wilson’s disease: an update

**DOI:** 10.1515/almed-2022-0020

**Published:** 2022-04-26

**Authors:** Eduardo Martínez-Morillo, Josep Miquel Bauça

**Affiliations:** Department of Laboratory Medicine, Complejo Asistencial Universitario de Salamanca (CAUSA), Salamanca, Spain; Department of Laboratory Medicine, Hospital Universitario Son Espases, Palma de Mallorca, Spain

**Keywords:** *ATP7B* gene, ceruloplasmin, copper, Wilson’s disease

## Abstract

Wilson’s disease (WD) is an inherited disorder of copper metabolism caused by mutations in the *ATP7B* gene. This condition is characterized by the accumulation of copper in the liver and other organs and tissues causing hepatic and neuropsychiatric manifestations. This paper reviews the diagnostic performance and limitations of the biochemical tests commonly used to detect this underdiagnosed disease. It also provides some recommendations and suggests a set of standardized laboratory comments. At present, a rapid, simple, reliable biochemical test that confirms diagnosis of WD is not available. However, diagnosis can be established based on serum ceruloplasmin and urinary copper excretion. Total serum copper should be employed with caution, since it has a low negative predictive value. The use of estimated non-ceruloplasmin-bound copper is not recommended. Nevertheless, measured relative exchangeable copper has very high sensitivity and specificity and emerges as a potential gold standard for the biochemical diagnosis of WD. The development of novel assays for WD detection makes this disorder a potential candidate to be included in newborn screening programs.

## Introduction

Wilson’s disease (WD) is an autosomal recessive disorder of copper metabolism caused by mutations in the *ATP7B* gene, which is located in the long arm of chromosome 13 (locus 13q14.3) [1, 2]. This gene encodes a copper-transporting ATPase expressed predominantly in the liver. This protein has six metal-binding sites and eight transmembrane domains, which form a pore for ATP-dependent copper transport through membranes [3]. The ATP7B protein is essential for the transport and biliary excretion of this trace element [4].

In patients with WD, copper stored in the hepatocytes cannot be eliminated or complexed to ceruloplasmin, the main copper-transporting protein, for subsequent release into the bloodstream [3]. This alteration results in excess concentration of copper, which initially accumulates in the hepatocyte and then in other organs and tissues, especially in the brain, which causes mitochondrial dysfunction and apoptosis. In addition, the formation of oxygen-reactive species and direct interaction of copper in lipid synthesis cause alterations in cell energy metabolism and the deregulation of genes involved in cholesterol biosynthesis [4]. Moreover, neurons, especially those located in basal ganglia, are more susceptible to the effects of excess copper deposition. This leads to the appearance of a wide variety of symptoms including neurological, psychiatric, ophthalmological and hepatic manifestations [3].

WD is mostly diagnosed in patients aged 5–35 years, although symptoms may appear at any age [5]. There are reports of cases diagnosed from 9 months to 77 years of age [6, 7]. Hepatic manifestations include asymptomatic hepatomegaly, isolated splenomegaly, intermittent or persistent transaminase elevation, jaundice, fatty liver, compensated acute hepatitis or decompensated cirrhosis, or acute liver failure. The most frequent neurological symptoms include dysarthria, postural tremor, dystonia, parkinsonism, ataxia and chorea. Around 2/3 of WD patients exhibit psychiatric symptoms at diagnosis and 20% develop them before diagnosis. The most common psychiatric manifestations include incongruous or antisocial behavior, irritability, personality changes, and depression. With regard to ophthalmological features, Kayser-Fleischer rings are found in most cases of WD with neurological presentation, whereas they are only observed in 50% of patients with hepatic presentation and in less than 30% of asymptomatic patients [3, 8]. Other less frequent clinical manifestations are hematological (non-autoimmune hemolytic anemia, coagulopathy, thrombocytopenia), renal (acute renal failure, nephrolithiasis, urolithiasis, renal tubular acidosis), musculoskeletal (arthropathy, muscle weakness) or other (heart disease, pancreatitis, hypoparathyroidism) [5].

The overall prevalence of WD is estimated to range between 1:10.000 and 1:30.000, with a rate of heterozygous carriers of 1:70 [9, 10]. To date, more than 700 pathogenic or probably pathogenic *ATP7B* mutations have been identified, including 279 variants associated with the loss of protein function [10]. Multiple studies demonstrate a poor genotype-phenotype correlation, with incomplete penetrance. Interactions between epigenetic and metabolic factors are known to contribute to the varying phenotypes of WD [11]. The different variants cause distinct abnormalities in copper homeostasis. The variants causing loss of protein function are associated with high penetrance, whereas missense variants are linked to lower penetrance. Thus, for a genetic diagnosis of WD, it is important that the two mutations detected are pathogenic. Moreover, other biochemical studies are required to establish the level of copper homeostasis dysfunction and help in therapeutic decision-making [10, 12]. Molecular diagnosis of WD involves entire *ATP7B* gene sequencing to detect pathogenic variants either by the gold-standard method (Sanger sequencing) or by parallel massive sequencing (Next-Generation Sequencing, NGS) [13]. The MLPA (Multiple Ligation-dependent Probe Amplification) technique can complement direct sequencing to detect large deletions and duplications. The main advantage of this study is that diagnosis of WD is confirmed when two pathogenic variants associated with the loss of protein function are detected. The main limitations are that it is a relatively expensive study and that interpretation of results may be challenging when variants of uncertain significance are identified [14]. Therefore, molecular diagnosis is usually limited to cases with high suspicion of WD based either on clinical or biochemical findings.

Early diagnosis is essential to prevent complications in the long term. Currently, WD remains an underdiagnosed condition. Delayed diagnosis is the most common cause of severe complications and mortality [15]. In contrast, early management of asymptomatic patients may prevent the occurrence of clinical manifestations [4]. However, to date, the majority of asymptomatic patients are diagnosed during familial genetic screening of WD subjects [5].

Diagnosis of WD is challenging due to the lack of a rapid, simple, reliable, accurate test that is pathognomonic of the disease. On average, the time elapsed between the onset of symptoms and diagnosis exceeds two years, with a greater delay in patients with a neurological presentation [8]. Patients with WD generally exhibit unspecific clinical manifestations, and diagnosis is established based on a combination of clinical and laboratory studies [16]. In addition, the presence of atypical manifestations also delays diagnosis [8]. WD may be misdiagnosed as a result of a poor interpretation of symptoms and/or laboratory results, thereby exposing the patient to unnecessary treatment and its potential side effects [17].

At present, most therapeutic strategies in Europe for WD include D-penicillamine, trientine or zinc salts as the first therapeutic options. The first two are chelating agents that help to solubilize copper in the cells to allow for more rapid urinary excretion, while zinc salts inhibit intestinal copper absorption. Of all these treatments, D-penicillamine is associated with a higher frequency of adverse events [18]. Finally, severe cases of WD may require a liver transplant [3, 5].

## Purpose and scope

This document is aimed at providing a review of the main biochemical tests available for the diagnosis of WD. To such purpose, we performed a review of the scientific evidence available on diagnostic performance, limitations and main causes of false positive (FPs) and false negative (FNs) results. This document is also intended to help interpret laboratory results and establish a more accurate diagnosis of WD. We provide some recommendations and suggest a set of standardized comments to be included in laboratory reports (Tables 1 and 2).

**Table 1:** Recommendations for the analysis and interpretation of ceruloplasmin and copper values in the diagnosis of Wilson’s disease (WD).

	Recommendation
**Pre-analytical ph** **ase**	

Ceruloplasmin	Do not determine this protein in very lipemic specimens
	Determination can be performed either in serum or plasma, but serum is recommended for simultaneous determination of ceruloplasmin and copper
Copper in blood	Plasma is not appropriate for evaluating nutritional status of copper or establishing diagnosis of WD
	Total serum copper concentration has very low negative predictive value for WD and is not recommended when screening for this disease
Urinary copper	24-h urinary copper is recommended. There is no solid scientific evidence supporting the use of random urine for the diagnosis of WD
Liver copper	Determination of intrahepatic copper is not recommended in patients with a cholestatic disease (such as primary biliary cholangitis or primary sclerosing cholangitis)

**Post-analytical phase**	

Ceruloplasmin	Upon suspicion of an acute phase process (i.e. inflammation), determination of another acute phase reactant (i.e. C-reactive protein) is recommended to identify potential false negative results
Serum copper	Upon a finding of low serum copper values in young patients without a known cause of copper deficiency, adding a ceruloplasmin test is recommended
	Upon serum copper concentrations >8 μmol/dL (500 μg/dL), consider sample contamination
Urinary copper	Urinary copper in patients with kidney dysfunction should be interpreted with caution, since there is a significant relationship between reduced glomerular filtrate and elevated urinary copper excretion. This test is not indicated in severe cases of kidney failure due to the high risk of obtaining a false positive result

**Biochemical markers**	

Non ceruloplasmin-bound copper	The use of formulas for quantification of non-ceruloplasmin-bound copper is not recommended
Relative exchangeable copper	This biochemical marker has a high diagnostic potential and should be evaluated in clinical laboratories

**Table 2:** Standardized comments for reporting ceruloplasmin and copper values in the diagnosis of Wilson’s disease (WD).

	Comment
**Pre-analytical phase**	

Very lipemic serum specimen	Ceruloplasmin cannot be determined in very lipemic specimens

**Post-analytical phase**	

Ceruloplasmin: <0.1 g/L	Ceruloplasmin and urinary copper concentrations are strongly suggestive of WD
Urinary copper: >1.6 µmol/24-h	
Ceruloplasmin: >0.2 g/L	Ceruloplasmin and urinary copper concentrations indicate that WD is unlikely
Urinary copper: <0.64 µmol/24-h (40 µg/24-h)	
Total serum copper: <0.8 μmol/dL (50 μg/dL)	Consider possible severe copper deficiency or WD
Total serum copper: >4 μmol/dL (250 μg/dL)	Consider possible life-threatening liver failure (it does not apply for pregnant women and women on estrogen treatment)
Total serum copper: >8 μmol/dL (500 μg/dL)	Send a new sample to rule out copper contamination

## Biochemical studies for the diagnosis of WD

A rapid, reliable, non-invasive test with high discriminative power is necessary for an accurate diagnosis of WD. However, most of the laboratory tests currently available have limitations and, when used alone, do not allow for the establishment of a final diagnosis of WD. Proper interpretation of results is essential for a correct diagnosis.

Biochemical features of WD generally include reduced concentrations of ceruloplasmin and total serum copper, elevated urinary copper excretion, and abnormally elevated levels of intrahepatic copper [1]. However, the classic triad, namely, low ceruloplasmin, low serum copper, and elevated urinary copper, may be absent in a significant number of WD patients and is found in more than 15% of heterozygous carriers [8].

### Serum ceruloplasmin

Ceruloplasmin, an enzyme with ferroxidase activity, is the major copper-carrying protein, and contains up to 70–90% of circulating serum copper. Ceruloplasmin is synthesized in the liver in the form of apoceruloplasmin, which is enzymatically inactive, where it incorporates 6–8 atoms of copper and activates to form holoceruloplasmin. Copper uptake by apoceruloplasmin and subsequent release into the bloodstream by the hepatocyte is ATP7B-dependent. If copper is not incorporated, apoceruloplasmin is rapidly degraded. Low concentrations of holoceruloplasmin in serum are a very specific biochemical characteristic of WD [4, 5].

Ceruloplasmin is commonly measured in serum (although it can also be measured in plasma) by immunoturbidimetric or immunonephelometric methods. These techniques measure holoceruloplasmin and apoceruloplasmin and help establish WD diagnosis or identify other copper-deficiency conditions. A recent meta-analysis reported a sensitivity of ceruloplasmin of 77–99%, with specificity for diagnosis of WD ranging from 56 to 83% (according to the different studies included). These estimations are based on a cut-off value of 0.2 g/L (lower limit of reference, LLR) established in the International Meeting on WD held in Leipzig in 2001 [19] and reported by most manufacturers. When the cut-off value is established at 0.1 g/L, sensitivity decreases to 65–79%, but specificity increases to 97–100% [16]. Given the unavailability of standard ceruloplasmin measuring methods, it is important to use the LLR reported by the manufacturer, as in the case of reagent CERU for Cobas^®^ (Roche Diagnostics), since the use of a cut-off value of 0.2 g/L with this assay may provide a different diagnostic performance.

Ceruloplasmin concentrations may increase in pregnant women or in women using oral contraceptives or estrogen treatments. In addition, as it is a positive acute phase reactant, it can also increase in the presence of inflammation, infection, rheumatoid arthritis, and in patients with myocardial complications or cancer (FNs). In addition, low serum ceruloplasmin concentrations (FPs) may be obtained in patients with acute viral hepatitis, drug-induced, alcoholic, or end-stage liver disease, malabsorption, malnutrition, cachexia, protein-losing nephropathy, acquired copper deficiency (e.g., following zinc poisoning), Menkes syndrome, aceruloplasminemia, or in healthy carriers (Table 3) [5, 8].

**Table 3:** Biochemical tests, diagnostic performance, and causes of false positive (FPs) and false negative (FNs) results in Wilson’s disease.

Test	Cut-off	Sensitivity	Specificity	Causes of FPs	Causes of FNs	References
Ceruloplasmin	<0.1 g/L	65–79%	97–100%	Viral acute hepatitis;drug-induced liver injury; alcoholic liver disease; terminal liver disease; malabsorption; malnutrition; cachexia; protein-losing nephropathy; acquired copper deficiency; Menkes disease; aceruloplasminemia; heterozygous carriers; lipemic specimens	Pregnancy; contraceptives; estrogens; inflammation; infection; arthritis; myocardial injury; cancer; immunoassay	[16]
	<0.2 g/L	77–99%	56–83%			
Urinary copper	>0.64 µmol/24-h (40 µg/24-h)	79%	88%	Autoimmune hepatitis; active chronic liver disease; cholestasis; acute liver failure; heterozygous carriers	Kidney failure; inadequate/incomplete sample collection	[35]
	>1.6 µmol/24-h (100 µg/24-h)	50–80%	76–97%			[16]
Liver copper	>4 μmol/g (250 μg/g)	66–94%	52–99%	Cholestasis	Heterogeneous deposit distribution	[16]
Relative exchangeable copper	14–18.5%	92–100%	99–100%	Not reported	Not reported	[29, 32, 47]

Total serum copper and calculated non ceruloplasmin-bound copper are not included in the Table, since their use is not recommended for the diagnosis of WD.

A limitation of ceruloplasmin determination is the widespread use of immunoassays. These methods measure both, holoceruloplasmin and apoceruloplasmin, and as a result, they indirectly overestimate the enzymatic activity of holoceruloplasmin. This has been observed when results have been compared with those obtained by methods based on the direct determination of enzymatic activity [8]. Therefore, normal concentrations of ceruloplasmin do not exclude the possibility of a low ferroxidase activity (FN). The specific enzymatic activity of ceruloplasmin is sensitive to copper status and is not dependent on age, sex or hormonal alterations [20]. Therefore, it is recommended that enzymatic methods be used for the diagnosis of WD. However, they are not usually available in clinical laboratories due to specimen instability and the lack of standardization [4, 5, 21]. Finally, samples with a high lipid content should not be used, since they may yield falsely low results (FP) [17]. Manufacturers do not usually warn about FP results, since interference studies are generally performed with Intralipid^®^, and this emulsion is not always representative of the complex composition of the blood samples of many patients with high triglyceride concentrations [22, 23].

### Serum copper

Copper is a trace element necessary for the proper functioning of multiple enzymes. Serum is the sample of choice to assess nutritional copper status in the body. The use of plasma is not recommended, as copper concentrations are lower in plasma than in serum and do not accurately reflect copper nutritional status [24]. Copper is determined in serum and urine by inductively-coupled plasma mass spectrometry (ICP-MS) or atomic absorption spectrometry. ICP-MS is the most widely used technology among participants in the OELM European Organization for External Quality Assessment Programme. The two techniques involve some preanalytical, technical and sample preparation considerations that were thoroughly discussed in a previous document of the Trace Elements Commission [25, 26].

In normal conditions, serum copper concentrations vary proportionally to ceruloplasmin concentration. Therefore, copper is generally reduced in WD. However, some WD patients exhibit normal or even elevated copper concentrations, which suggests the presence of a high volume of circulating non-ceruloplasmin-bound copper [5]. Some clinical conditions may cause the elevation of serum copper concentrations, regardless of ceruloplasmin concentrations, including acute liver failure of any cause, due to the profuse release of copper from the liver, chronic cholestasis or, less frequently, copper poisoning (FNs). Elevated copper concentrations in women of childbearing age usually are associated with pregnancy or the use of oral contraceptives or estrogen treatments. More rarely, reduced copper concentrations (FPs) are found in patients with acquired copper deficiency (secondary to gastric bypass, gastrectomy or excess intake of zinc, among other causes), in Menkes disease, and in a low percentage of heterozygous carriers [4, 27].

Although it is customary in clinical practice, several national and international associations such as EASL, AASLD, AEEH or APASL do not recommend using total serum copper for the diagnosis of WD, primarily for its low negative predictive value, with specificity ranging 94–100% but with a sensitivity of 69–74% [27], [28], [29]. Therefore, normal serum copper concentrations do not exclude WD. However, abnormally low copper concentrations are strongly suggestive of WD, given the high positive predictive value of the test, once other potential causes of copper deficiency have been ruled out. In the presence of low copper concentrations, especially in young patients, determination of serum ceruloplasmin is recommended.

The reference interval for serum copper concentrations is 1.1–2.5 μmol/dL (70–155 μg/dL) [25], with small variations according to age and sex, especially in pregnant women or on estrogen treatment, who may show concentrations of up to 4.75 μmol/dL (300 μg/dL) [20]. Concentrations <0.8 μmol/dL (50 μg/dL) indicate severe copper deficiency or WD [29], [30], [31], [32]. Concentrations >4 μmol/dL (250 μg/dL) are rarely found and may suggest fulminant hepatic failure [28, 33]. Values >8 μmol/dL (500 μg/dL) are very infrequent and may suggest sample contamination.

### Urinary copper

In patients with WD, deficient biliary copper excretion leads to urinary excretion, thereby increasing urine copper concentrations. Although a cut-off value has not been unequivocally established in children, it is recommended to use a lower threshold in pediatric patients, due to the reduced time of copper accumulation in the body [34].

According to the literature on pediatric patients, 24-hour (24-h) urinary copper concentrations have a sensitivity of 79% and a specificity of 88% for the diagnosis of WD when a cut-off value of 0.64 µmol/24-h (40 µg/24-h) is used, and a sensitivity of 50–80% and a specificity of 76–97% for a cut-off value of 1.6 µmol/24-h (100 µg/24-h) [16, 35]. Despite the limitations associated with the collection of 24-h urine, copper determination in random urine samples is not recommended due to large within-subject variability [25]. Some authors suggest using urinary copper/creatinine or copper/zinc ratios for the diagnosis of WD in children. However, there is no solid evidence supporting this practice nor have unequivocal reference values been established [36, 37].

The interpretation of urinary copper concentrations may be challenging, especially in patients with kidney failure, since patients with reduced glomerular filtrate show higher urinary copper concentrations [38, 39]. In other cases, urine sample may be insufficient or incomplete (FNs). Elevated urinary copper concentrations are also found in other liver diseases (FPs), including autoimmune hepatitis, active chronic liver disease or cholestatic syndromes, and particularly during acute liver failure of any type. In heterozygous carriers, copper concentrations often fall between the ones observed in WD patients and those found in healthy patients or they may even exceed the upper limit of reference (ULR) (Table 3) [5, 8].

### Liver copper

Liver biopsy is an invasive technique that involves some risks; consequently, it is not generally recommended in asymptomatic or pediatric patients. This technique is used in adult patients with clinical or biochemical suspicion of WD, generally with a hepatic presentation, but without a final diagnosis having been established. Liver biopsy is rarely needed in patients with a neuropsychiatric presentation [3, 5].

Specimens need to be processed under specific conditions, according to the Clinical and Laboratory Standards Institute (CLSI) guidelines. It is essential that an adequate sample size is taken (not less than 5–10 mm). The tissue needs to be irrigated with deionized water (never use a saline solution) and poured into a polypropylene tube without adding water or any other fluid. The container has to be properly closed and sent to the laboratory within 24-h after collection. Otherwise, it has to be stored at −20 °C [40].

According to the literature, liver copper has a sensitivity of 66–94% and a specificity of 52–99% for the diagnosis of WD when a cut-off value of 4 μmol/g of dry weight (250 μg/g) is used. The main limitations of liver copper determination are variability in the distribution of intrahepatic copper deposits, which may yield a FN result if the sample collected is not representative, and the elevation of copper deposits in patients with cholestatic disease (FPs) (Table 3). Therefore, factors such as the required sample size and the optimal cut-off value are a matter of debate [5, 16, 41].

### Serum non-ceruloplasmin-bound copper (NCC)

Calculated non-ceruloplasmin-bound copper (NCC) has been proposed as a diagnostic marker of WD, since serum concentrations are theoretically elevated in patients with the disease. Concentrations can be calculated using the following formula:

NCC (µmol/L) = total copper (µmol/L) − 49 (µmol/g) × ceruloplasmin (g/L)

Forty-nine is the factor from which ceruloplasmin-bound copper concentration (in µmol/L) is estimated, based on serum ceruloplasmin concentration (in g/L).

The usefulness of this marker has been the subject of intense controversy. Some studies reveal a 25% of FNs in patients with WD when ceruloplasmin is determined by immunoassay. Moreover, NCC may be elevated (FPs) in patients with cholestasis, acute liver failure or copper poisoning [5]. On the other hand, estimation of this parameter is not transferable between laboratories, and values < 0 may be obtained in more than 20% of patients, which is physiologically impossible. For these reasons, as it occurs with serum copper, the European Association for the Study of the Liver (EASL) does not recommend the use of ceruloplasmin-bound copper concentration for the diagnosis of WD [27, 42, 43].

### Exchangeable copper (CuEXC) and relative exchangeable copper (REC)

In the last years, several studies have assessed the diagnostic performance of direct determination of exchangeable copper (CuEXC) in serum of patients with WD, with encouraging sensitivity and specificity results [8].

Between 70 and 90% of circulating copper is ceruloplasmin-bound, with a small proportion bound to albumin (≈15%), 2-macroglobulin (≈10%), amino acids or circulating freely in the bloodstream (<5%). Hence, CuEXC is thought to correspond to the labile fraction of copper complexed to albumin, but also to *α*2-macroglobulin, amino acids, and the external structure of ceruloplasmin, and represents bioavailable copper for most of cells owing to this labile binding [29, 44, 45].

CuEXC determination involves simple incubation of serum in an EDTA solution for 1 h, and subsequent ultrafiltration of diluted serum [29]. Nevertheless, the marker with the highest diagnostic power for WD is relative exchangeable copper (REC):

$$\text{REC}\left(\text{\%}\right)=\left(\text{CuEXC}/\text{total copper}\right)\times 100$$

where total copper concentration is obtained from original serum and CuEXC is obtained from ultrafiltered serum after sample processing.

The diagnostic performance of REC reported by El Balkhi et al. was 100% in the detection of WD (cut-off: >18.5%), when results were compared with those of 62 healthy subjects, 25 members of the family of WD patients and without mutations in *ATP7B*, and 45 heterozygote carriers [29]. In a later study, Trocello et al. observed that REC discriminates WD patients (homozygote or double heterozygote) from heterozygote carriers. All WD patients exhibited a REC >15%, whereas none of the other participants exceeded this threshold [32]. Poujois et al. also evaluated CuEXC concentrations in patients with WD and various forms of clinical presentation, namely, pre-symptomatic, with hepatic presentation and with extra-hepatic presentation. The authors found that CuEXC was significantly more elevated in the patients with an extra-hepatic presentation, which suggests that this parameter could be a marker of disease severity [46].

REC has also demonstrated a very high diagnostic performance in studies comparing WD patients with patients affected with other liver diseases, with a sensitivity of 100% and a specificity of 99% using a cut-off of 14%; and a sensitivity of 92% (four cases of treated patients with WD were not detected) and a specificity of 100% for a cut-off of 18.5% [47].

CuEXC and REC may provide useful information about the severity and level of spread of WD, showing a promising diagnostic performance. Moreover, reference values for CuEXC and REC have been recently published for the 1–18 year population [48, 49]. However, prospective studies that confirm the reliability of this biochemical parameter are necessary. Nevertheless, encouraging results have been obtained to date and support the use of this test in selected cases to identify the patients who may benefit from screening for *ATP7B* mutations. Although CuEXC determination increases testing costs by about 10 euros (with a total cost < €20), it is substantially below the cost of genetic sequencing, which cost is €500–1,000.

### Other biochemical tests

Since FN results are usually obtained for ceruloplasmin and total serum copper concentrations in patients with fulminant liver failure secondary to WD, which hinders diagnosis, the potential of other biochemical parameters to identify these patients has also been assessed. Korman et al. reported that, concurrent to other symptoms, an alkaline phosphatase (ALP)/total bilirubin ratio <4 has a sensitivity of 94% and a specificity of 96% for the diagnosis of WD, whereas an AST/ALT ratio >2.2 has a sensitivity of 94% and a specificity of 86% [28]. More recently, a scoring system based on AST, ALT, ALP, AST/ALT ratio, urate and hemoglobin results has been documented to have a sensitivity of 88% and a specificity of 87% for the diagnosis of fulminant WD [33]. However, these tests have a significantly lower diagnostic performance in the pediatric population [50].

## Diagnostic criteria for WD

As described above, with the exception of CuEXC and REC (still under study), none of the biochemical tests used in routine practice allows for an accurate diagnosis of WD. Several scientific societies have published clinical guidelines with different diagnostic criteria. The American Association for the Study of Liver Diseases (AASLD) proposed a clinical/biochemical algorithm for the diagnosis of WD. EASL and the European Society for Paediatric Gastroenterology Hepatology and Nutrition (ESPGHAN) advocate the use of the Leipzig scoring system (Table 4) [19].

**Table 4:** Leipzig scoring system for the diagnosis of Wilson’s disease.^a^

Symptoms, signs and tests (score)	
**Serum ceruloplasmin**	**Kayser-Fleischer rings:**

>0.2 g/L: 0 points	Present: 2 points
0.1–0.2 g/L: 1 point	Absent: 0 points
<0.1 g/L: 2 points	

**Urinary copper^b^**	**Neurological symptoms:**

Normal: 0 points	Severe: 2 points
1–2× upper limit of reference: 1 point	Mild: 1 point
>2× upper limit of reference: 2 points	Absent: 0 points

**Intrahepatic copper^c^**	**Mutation analysis** **^d^:**

>4 μmol/g (>250 μg/g): 2 points	On both chromosomes: 4 points
0.8–4 μmol/g (50–250 μg/g): 1 point	On one chromosome: 1 point
<0.8 μmol/g (<50 μg/g): −1 point	No mutations: 0 points

**Coombs-negative hemolytic anemia**	**Overall score:**

Present: 1 point	≥4 points. Diagnosis established
Absent: 0 points	3 points. Likely diagnosis
	≤2 points. Very unlikely diagnosis

^a^Modified from (19). ^b^In absence of acute hepatitis. ^c^In absence of cholestasis. ^d^Detection of pathogenic or probably pathogenic variants.

The algorithm proposed by AASLD is based on an initial ophthalmological screening for Kayser-Fleischer rings and on serum ceruloplasmin and urinary copper determination. This algorithm varies depending on whether the patient shows neuropsychiatric and/or hepatic symptoms. When inconsistent results are obtained, a liver biopsy or genetic testing is recommended. In contrast, the diagnostic strategy recommended by EASL and ESPGHAN is based on the Leipzig scoring system and genetic testing rather than on liver biopsy, to avoid invasive procedures [19, 27, 51, 52]. The main limitations of the scoring system is that it is based on the opinions of a panel of experts instead of population-based studies, added to the lack of a standard ULR for urinary copper excretion. Nevertheless, the Leipzig scoring system has been evaluated in pediatric patients with satisfactory results, with a sensitivity of 93–98% and a specificity of 92–97% [5, 35, 53].

**Figure 1:**
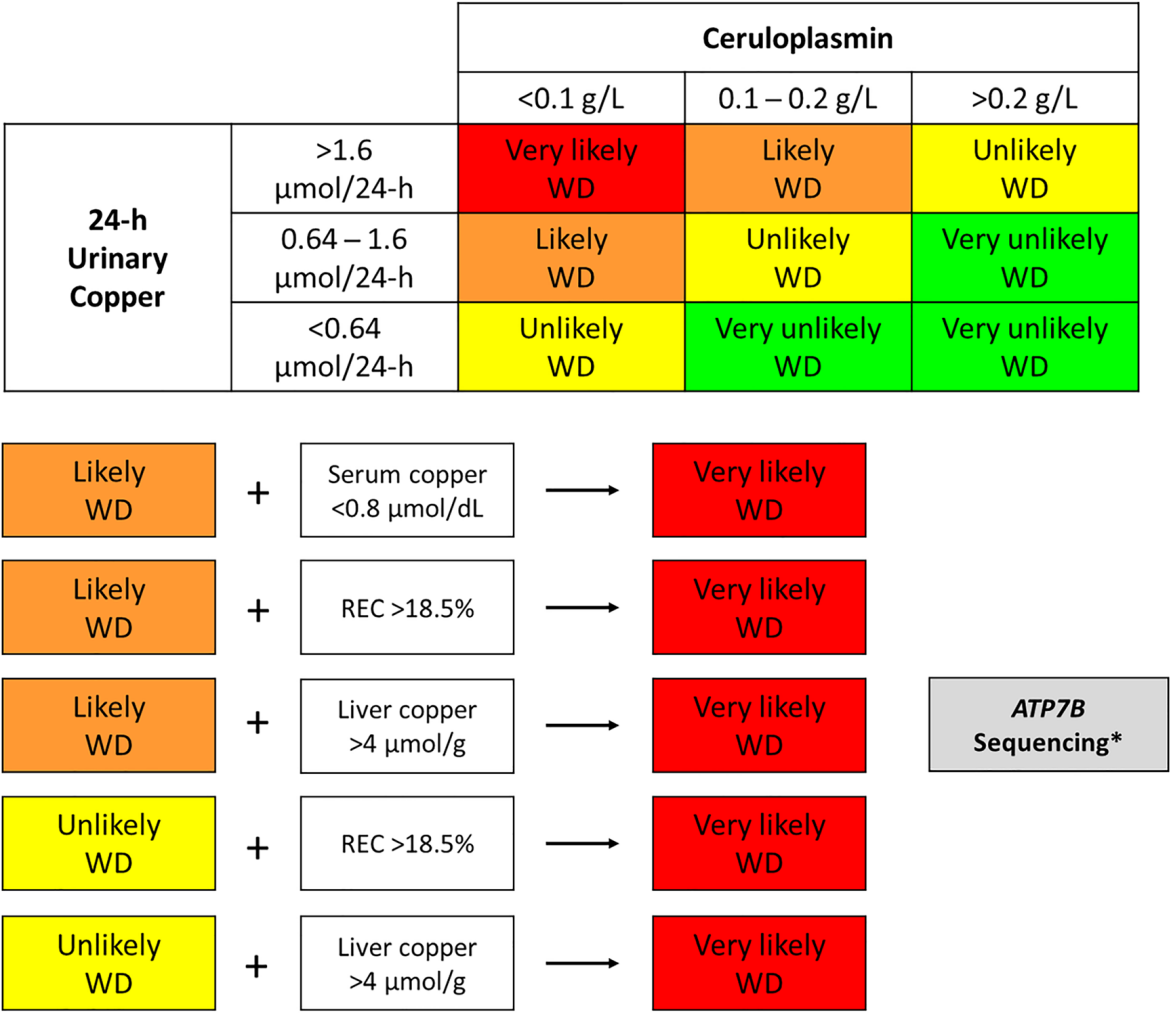
Algorithm for biochemical diagnosis of Wilson’s disease (WD). Total serum copper concentration <0.8 μmol/dL in the presence of intermediate ceruloplasmin concentrations (0.1–0.2 g/L) grounds suspicion of a very likely case of WD (because total serum copper has a high positive predictive value). A relative exchangeable copper (REC) result >18.5% or intrahepatic copper concentrations >4 μmol/g in the absence of cholestasis is suggestive of a very likely case of WD. *Genetic testing is strongly indicated in patients with laboratory results suggestive of a very likely case of WD or in patients with laboratory results suggestive of likely WD in whom a liver biopsy is to be avoided.

Considering the aforementioned, we propose an algorithm for the biochemical diagnosis of WD based on laboratory test results (Figure 1).

## Newborn screening for WD

WD meets most of the criteria proposed by Wilson and Jungner, which are used as a reference to determine the diseases to be incorporated in newborn screening programs. WD is a major health problem with a known natural course involving a long period of latency where the patient remains asymptomatic, for which effective treatments are available to prevent typical manifestations. However, WD has not yet been incorporated in newborn screening programs owing to the unavailability of a cost-effective test that allows for its detection. The studies assessing ceruloplasmin as a screening test for WD have been unsuccessful. These studies report a significant proportion of FNs due to ceruloplasmin being a positive acute phase reactant, and a high frequency of FPs, since a substantial number of neonates show low physiological ceruloplasmin concentrations [54], [55], [56], [57].

A method has been recently developed by which ATP7B peptides in dried blood spot samples (heel prick test) are measured by tandem mass spectrometry after antibody-based enrichment. This method has a sensitivity of 91% and a specificity of 98% and shows promise as an avenue for including WD in newborn screening within the coming years [58, 59].

## Conclusions

The biochemical tests available for diagnosis of WD have limitations that render diagnostic performance suboptimal. Promising data have been reported for CuEXC and REC, which encourages laboratory specialists to incorporate and evaluate these parameters. Although genetic tests for diagnosis of WD are increasingly available, the absence of a genotype-phenotype correlation requires the use of other markers to assess copper metabolism in these patients. Finally, newborn screening for WD may become reality in the near future if a cost-effective method is developed.

### Appendix

Members of the Commission (in alphabetical order): J.M. Bauça Rosselló, P. Bermejo Barrera, A. Bravo Gómez, J.A. Cocho de Juan, M. González Estecha (Chair since March 2019), J. González Revaldería, S. Izquierdo Álvarez, M.T. Llorente Ballesteros, E. Martínez González, E. Martínez Morillo, J.P. Sánchez Marín, S. Pérez San Martín and E. Urrechaga Igartua.
